# Silicone Gel for Hypertrophic Scars

**DOI:** 10.4103/0974-2077.69031

**Published:** 2010

**Authors:** Viroj Wiwanitkit

**Affiliations:** *Wiwanitkit House, Bangkhae, Bangkok - 10160, Thailand. E-mail: wviroj@yahoo.com*

Sir,

I read with great interest the report by Puri *et al*. on the usage of silicone gel for the treatment of hypertrophic scars.[1] Puri *et al*. mentioned that “The results of the self-drying silicone gel have been satisfactory.[1]” Indeed, silicone gel is proved to be an effective choice. de Giorgi *et al*. reported that “silicone gel is able to reduce the formation of keloid and hypertrophic scars and the signs/symptoms associated with the healing process (paraesthesia, pulling sensation, alterations in colour.[2]” However, there are some points that need to be discussed. There is no control group in this work. Hence, it cannot imply any comparative effectiveness to other alternative treatments. Karagoz *et al*. recently performed a comparative study among silicone gel, silicone gel sheeting and topical onion extract, including heparin and allantoin, for the treatment of postburn hypertrophic scars and concluded that “Silicone products, either in gel or sheet, are superior to Contractubex in the treatment of the hypertrophic scar.[3]” Karagoz *et al*. also mentioned that “The therapist should select the most appropriate agent according to the patient’s need and guidelines of these signs.[3]” Further, comparative cost-effectiveness studies among various alternative choices should also be performed to obtain the data for decision-making for selection of the most appropriate method.
